# Factors influencing the admission decision for Medical Psychiatry Units: A concept mapping approach

**DOI:** 10.1371/journal.pone.0221807

**Published:** 2019-09-17

**Authors:** P. J. Caarls, M. A. van Schijndel, G. van den Berk, A. D. Boenink, D. Boerman, J. G. Lijmer, A. Honig, M. Terra, A. Thijs, B. Verwey, J. A. van Waarde, J. van Wijngaarden, J. J. van Busschbach, on behalf of the MPU Study Group

**Affiliations:** 1 Erasmus Medical Center, University Medical Center, Rotterdam, The Netherlands; 2 Rijnstate Hospital, Arnhem, The Netherlands; 3 Onze Lieve Vrouwe Gasthuis, Amsterdam, The Netherlands; 4 Amsterdam University Medical Center, Amsterdam, The Netherlands; 5 Erasmus School of Health Policy and Management, Erasmus University, Rotterdam, The Netherlands; Institute of Mental Health, SINGAPORE

## Abstract

**Objective:**

Medical Psychiatry Units (MPUs), also known as Complexity Intervention Units (CIUs), provide care for complex patients suffering from both psychiatric and physical disorders. Because there is no consensus on the indications for admission to an MPU, daily practice and effectiveness research are hampered. This study therefore used a concept mapping approach to investigate which organizational and medical factors determine the decision to admit a patient to an MPU.

**Methods:**

The first step of the concept mapping approach was to create a list of factors determining MPU admission from literature. Secondly, clinical experts sorted and ranked these factors. The sorted and ranked data were then analyzed, and a draft conceptual framework was created. A final conceptual MPU admission framework was then drawn during an expert consensus meeting and recommendations for implementation were suggested.

**Results:**

Thirteen clinical experts defined 90 factors from literature, which were sorted and ranked by 40 experts from 21 Dutch hospitals. This concept mapping approach resulted in a five-cluster solution for an MPU admission framework based on: 1. Staff competencies and organizational pre-requisites; 2. Patient context; 3. Patient characteristics; 4. Medical needs and capabilities; and 5. Psychiatric symptoms and behavioral problems. Furthermore, three inclusion and two exclusion criteria were formulated to help the clinicians decide whether or not to admit patients to an MPU. These criteria can be implemented in daily practice.

**Conclusion:**

Implementing the five criteria derived from this conceptual framework will help make the admission decision for complex patients with psychiatric and physical disorders to an MPU more correct, consistent, and transparent.

## Introduction

High acuity Medical Psychiatry Units, also known as Complexity Intervention Units (CIUs), [1], cater for patients that are too psychiatrically ill to be treated on medical wards in conjunction with consultation-liaison psychiatry and too medically ill to be on a conventional psychiatric inpatient service [2]. The underlying common purpose of all MPUs is that these are geared towards both the medical and psychiatric care needs of a patient, thus providing integrated care to the ‘whole’ patient [3]. Goals of these MPUs include improving physical and psychiatric care, reducing the stigma of psychiatric disorders, and increasing the effectiveness and cost-effectiveness of inpatient stays by decreasing length of stay and readmissions [4–6]. The term MPU is generic and as such allows for extensive medical and organizational variation [7]. In practice, this results in considerable uncertainty about treatment options and referral criteria among patients, their caregivers, referrers, and health insurers.

MPUs have existed in North America for 25–30 years [8]. A recent study found 175 such clinical inpatient units for integrated medical and psychiatric care (van Schijndel et al 2019, in preparation). In Europe, similar programs were implemented in the 1990s [8–11]. In the Netherlands, an evolution has recently been taking place from general hospital psychiatry inpatient wards to MPUs [12]. This development was enhanced by the establishment of consensus-based Dutch quality standards for MPUs ([13, 14] and S1 Appendix) and a change in the reimbursement as well as procurement policies of health insurers, focusing on integrated medical and psychiatric care as of 2012. A recent study in the Netherlands showed that of the 90 Dutch hospitals in 2015, 37 had a ward for integrated medical and psychiatric care [12]. Of these units, 30 were classified as MPUs [15].

To date, there has been no research on the clinical reasons for admitting a patient to an MPU. It is unknown which medical and organizational factors may influence this clinical decision. Admission criteria for MPUs may depend on the target population, the focus on acute or chronic care, and both general medical and psychiatric care abilities of the specific unit. Some MPUs that have been described in literature reported detailed admission criteria, including criteria for continued stay and discharge [16, 17], while other MPUs reported a general target population [6] and still others reported no criteria [18, 19]. The lack of consensus on admission criteria hampers proper descriptions of MPUs, communication about treatment options, and studies about their effectiveness and cost-effectiveness. Consensus about inclusion and exclusion criteria for admitting a patient to the MPU may allow for more meaningful comparisons between MPUs in different settings [20, 21]. The Dutch context offers an opportunity to empirically elucidate the factors that influence admission decisions thanks to the high ‘MPU-density’ in the Netherlands and a clear consensus-based description of medical and organizational standards (Dutch quality standards for MPUs). The aim of this study, therefore, is to define which medical and organizational factors relate to MPU admission decisions.

## Method

### Concept mapping method

Concept mapping can provide insight into initially unclear complex decisions [22–24]. The concept mapping method is a standardized procedure used to clarify, describe, and visualize underlying cognitive structures of a task, such as medical decision making. The method uses both qualitative methods (group discussion) and quantitative methods (multidimensional scaling and cluster analyses) in a stepwise manner.

We chose the concept mapping methodology because it is an efficient way to explicate, structure, and prioritize tacit knowledge about topics on which the research literature is inconclusive [25]. In this study, we used it to explore the clusters of factors that influence the decision to admit a patient to an MPU. The results could be of value to patients, clinicians, and the scientific field. Improved referral could yield better outcomes by improved matching of care needs and the actual care that is provided. Understanding the relevant factors in admission decisions to MPUs may lead to more transparency in the care capabilities (and lack thereof) of MPUs, which is relevant from a referrer’s perspective, but also from a quality perspective because transparency will facilitate debate and discussion about desired capabilities versus the actual ones. Gaining insight into the implicit factors that currently guide the admission decision can also be relevant in advancing the evidence base on MPUs, by clarifying which properties of MPUs are relevant when comparing their outcomes in future studies.

The method consists of six phases that are summarized below. In this project, phase six was only partly carried out because we clearly recommended the use of the concept map and its further utilization is beyond the scope of this article. An extensive description of the six phases can be found in Trochim 19989 [26]. We sought informed consent from all the participants. Because there were no patients involved in the study and completion of the online task was not burdensome for the participants, Institutional Review Board approval was not required under Dutch law (Medical Research Act, article 1b).

#### Participants

A ‘core group’ of Dutch MPU experts was formed to guide the process of preparation, statement generation, interpretation, and utilization of the concept maps (see phase 1, 2, 5, and 6 below). Subsequently, a more extensive ‘expert group’ of MPU clinicians was formed for the actual sorting task (see phase 3 below). The following criteria were used to select the core group members: authoritative MPU clinicians (both psychiatrists and their somatic medical specialist coworkers) with a clinical as well as scientific background with diverse affiliations; from both academic and teaching hospitals and from MPUs with different organizational embeddings (internal medicine as well as psychiatry-based MPUs). Core group participants were selected from the professional network of the first author, with the addition of the only Dutch professor of General Hospital Psychiatry (AH), a psychiatrist representing the Dutch Psychiatric Association (BV), an organizational expert (JvWi), and a professor in Health-related quality of life (JB) with prior experience in concept mapping.

In addition, an expert group of psychiatrists and somatic specialists was recruited to participate in the study. In previous research studies, all MPUs in the Netherlands were assessed and their compliance with Dutch quality standards for MPUs was investigated [12]. MPUs could participate in this study if they complied with at least five of the ten MPU norms (30 out of 37 MPUs) defined in the quality standard. All psychiatrists that participated in our previous research [12] were also asked to participate in this study. Of the 30 psychiatrists that were approached, 20 agreed to participate, with the most common reason mentioned to decline being lack of time. Furthermore, the psychiatrists were asked to provide contact details of two or three of their most important somatic specialist coworkers. 37 somatic medical specialists were sent an invitation to participate, resulting in the participation of 21 somatic medical specialists.

#### Phase 1: Preparation

The focus question formulated by the core group was: “Which factors contribute to the decision of physicians to admit a patient to an MPU?”

#### Phase 2: Generating statements

The aim of this stage was to generate an exhaustive list of potential factors that represent the entire conceptual domain of the MPU admission decision [26]. Two methods were used: 1) a literature search and 2) experts’ opinions. First, a systematic literature search was conducted to gather all available literature on MPUs up to May 2014. Peer-reviewed articles in English describing an inpatient hospital ward aimed at diagnosing and treating a broad group of patients with active medical and psychiatric disorders were included. Based on title and abstract, 93 of the 4591 records were included and 78 full text articles were retrieved so that three of the authors (PC, MvS, and JvWi) could search for relevant factors. These factors were checked for overlap, relevance, and correct level of abstraction by four authors (PC, MvS, JvWi, and JvB). New articles were checked for factors until saturation of the factor list was achieved. Saturation occurred at 58 articles as the additional 20 articles that were checked for factors did not contribute further to the factor list. Secondly, all experts of the core group were asked to check the selected statements, add statements to the factor list, and remove irrelevant factors or change statements, but not to discuss the added value of each factor.

#### Phase 3: Structuring the statements: sorting and rating

All members of the expert group were invited to participate in the sorting and rating of the statements, which they could carry out on a personalized web application (www.conceptsystemsglobal.com). Participants were first asked five background questions, including their specialism, years of experience, and the type of hospital they worked in. The participants were then asked to group the factors influencing the admission decision in a way that made sense to them. Subsequently, they had to name each group based on a common characteristic. Sorting restrictions were: 1) factors could not be sorted according to importance; 2) not all factors could be placed in one group, nor could a group consist of only one factor; and 3) groups could not be named ‘other’ or ‘miscellaneous’. Participants were then asked to rate the importance and the commonness of each factor on a scale of 1 to 7. We emphasized that the factors could have a positive and/or a negative impact on the admission decision.

#### Phase 4: Statistical analyses and representation of statements

To analyze the results of the sorting and rating, four steps were taken. First, the individual sorting responses were represented in ‘individual binary similarity matrices’. The number of rows and columns in these matrices are the same as the number of factors (90). If a participant sorted two factors together in one cluster, a matrix cell was given the value (1); otherwise it was (0). The sum of all individual matrices resulted in a ‘combined within group similarity matrix’. This matrix represents the degree of similarity between a pair of factors over all participants. Secondly, a nonmetric multidimensional scaling (MDS) procedure on this ‘combined within group similarity matrix’ was used to represent each factor as a point on a two-dimensional map. The smaller the distance between points on the MDS map, the more frequently factors had been sorted together by the participants. The relationship between the distances on the point map and the similarities found in the original data was calculated in a stress value of the MDS map. The stress value can range from 0 to 1, where a higher score indicates a weaker relationship between the created point map and the original data. Thirdly, clusters of factors were constructed using a hierarchical cluster analysis according to Ward’s algorithm [27]. The average and median number of clusters made by the participants were checked. Furthermore, the clusters were checked for their bridging value, where a high value (near 1) indicates that the factors in that cluster were often sorted with factors from other clusters. In order to create distinct clusters, clusters with lower bridging values are preferred. A particular number of clusters is called a cluster solution; an optimum cluster solution has distinct and clinically relevant clusters. Lastly, the factors with the highest scores for importance and commonness were looked up.

#### Phase 5: Interpretation of the concept map

The core group was invited to determine the final concept map by discussing the appropriate number of clusters, the division of factors over the clusters, and the most appropriate names for the clusters.

#### Phase 6: Utilization of the concept map

How to implement the final concept map for use in daily hospital practice was discussed during the interpretation session.

## Results

### Participants

The expert group consisted of 20 psychiatrists and 21 other medical specialists from 21 hospitals, the response rate was 62%. Table 1 confirms that we were able to include a diverse group of appropriate participants: from all relevant specialties, from different hospital settings, and mainly clinicians who were very experienced clinicians and responsible for both referral and admission decisions to MPUs in daily practice.

**Table 1:** Characteristics of the 41 participants.

	N	%
Gender, female	10	24%
Specialism		
A. Psychiatry	20	49%
B. Internal Medicine	8	20%
C. Neurology	5	12%
D. Surgery	3	7%
E. Other medical specialty	5	12%
Type of hospital		
F. University Medical Center	10	24%
G. Teaching, top clinical hospital	25	61%
H.General hospital	6	15%
Years of experience with comorbidity		
I. 0–3 years	5	12%
J. 4–6 years	5	12%
K. 7–10 years	4	10%
L. More than 10 years	27	66%
Referrer and/or decision maker for admission		
O. Only referrer	8	19.5%
N. Referrer and decision maker	24	58.5%
O. Only decision maker	8	19.5%
P. Neither referrer nor decision maker	1	2.4%

### Generation of statements

A total of 58 articles (S2 Appendix) from the literature study were included and each article contributed between 4 and 274 factors. A list of 154 factors was distilled from these, sent to the core group, and discussed in the first meeting (March 2014). The excluded factors, which were deemed irrelevant or of an incorrect level of abstraction, were also sent to the core group to be checked. The core group stated that factors describing fixed MPU characteristics (i.e. total number of beds at the unit) should be excluded because these factors are irrelevant for admission decisions for individual patients. After removing these static factors, a list of 117 factors remained. During the second meeting (May 2014), a decision was made about whether specific physical symptoms or disorders should be included or combined. Several factors were removed, combined, or renamed, which resulted in a list of 77 factors. During the third meeting (August 2014), it was decided that psychiatric symptoms should be separated from psychiatric disorders, which resulted in an expanded list. A sorting and rating pilot session with field experts from one of the hospitals resulted in three additional factors (September 2015). The final list consisted of 90 factors and is shown in S3 Appendix.

### Sorting and rating of the factors

Forty participants took part in the sorting procedure, however, the sorting by two of the participants was excluded because their sorting violated the restrictions set a priori (do not sort based on importance and do not put all factors in one group) and technical and time constraints hampered making improvements. The 38 participants made a median of 7 clusters (average 7.8 clusters, standard deviation (SD) ±3.0, range 3 to 17). The rating of the importance of the factors was completed by 41 participants. On a scale of 1 to 7, the average importance was 4.8± 0.7, (range 2.8–6.7) suggesting moderate importance. The physicians stated that the most important factors were: competent nurses available on the MPU to meet patients’ care needs; medical facilities and skills on MPU, i.e. competent physicians available on the MPU to meet patients’ care needs; and behavioral or psychiatric problems that hamper other medical treatment. The rating of commonness of the factors was completed by 38 participants. On a scale of 1 to 7, the average commonness was 4.4± 0.7 (range 2.9–5.9). The factors that had the greatest impact on the admission decision were: patient needs somatic specialist care; number of MPU beds available at the time of referral/patient admission to the MPU; and competent nurses available on the MPU to meet patients’ care needs.

### Statistical analyses and representation

The nonmetric multidimensional scaling (MDS) procedure was used to draw a point map using the online Concept Systems Global software. Points that lie close together indicate that these factors were often sorted together. The stress value of the MDS map was 0.16, indicating a strong relationship between the distances on the point map and the similarities found in the original data. A point map drawn using only the psychiatrists input was relatively similar to the point map using the input of only non-psychiatrist physicians. At the interpretation session, various cluster solutions where shown to the experts, e.g. 4 cluster solutions, 5 cluster solutions, 6 cluster solutions, etc. They concluded that in shifting from five to six clusters, some factors were distributed over different clusters while they actually belonged together. The sixth cluster would also have an unfavorably high bridging value (0.94), which means that the factors in that cluster are often sorted with factors from other clusters. On the other hand, moving back from five to four clusters, factors on patient context and patient characteristics were clustered together, while the core group stated that they may have a different meaning in clinical practice.

### Interpretation session

**Fig 1:**
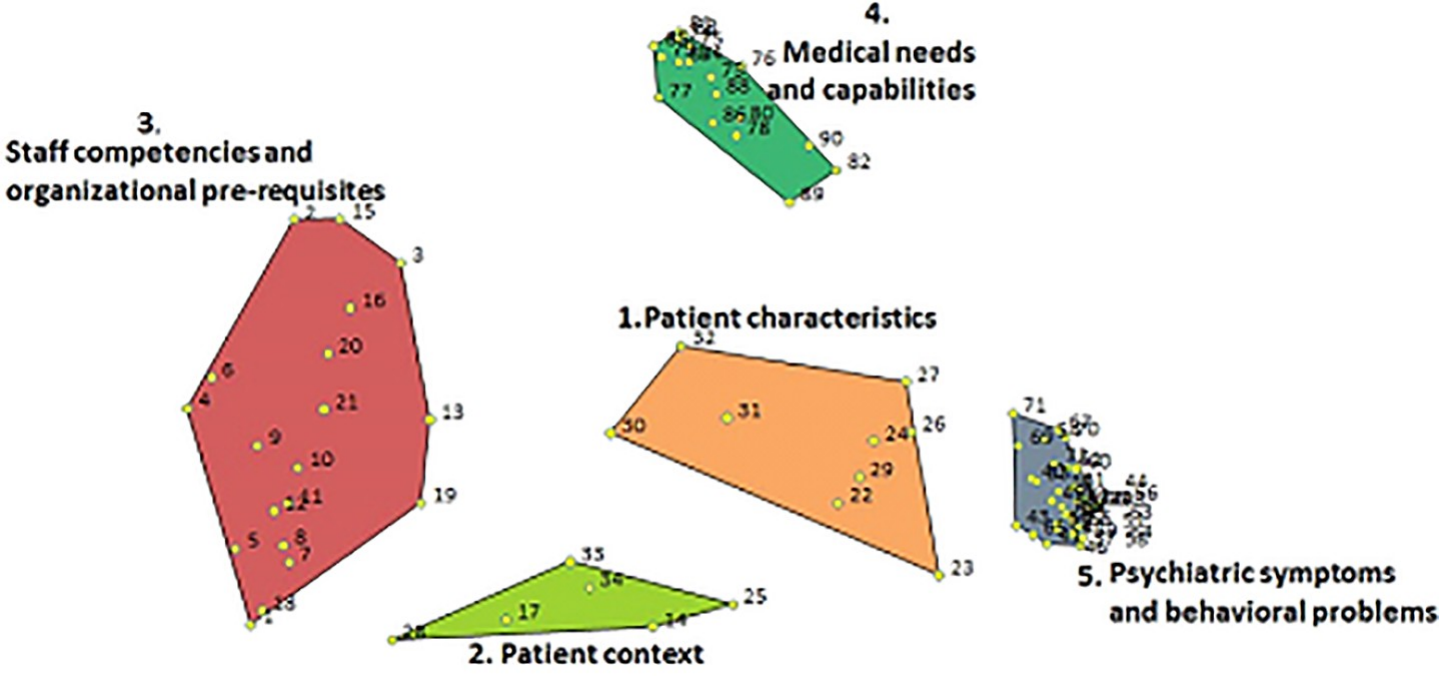
The 5-cluster solution.

An interpretation session with the core group was held in December 2017. The eight participants decided on the number of clusters, named them, and briefly described the different clusters. Fig 1 shows the final cluster map, starting at the center (1. Patient characteristics) and then clockwise, from the bottom. Clusters that lie closer together contain factors that were more often sorted together, which means that these clusters are more likely to be related. Clusters that lie further apart describe more distinct aspects of the decision-making process. The full list of factors and clusters is shown in S3 Appendix.

The five clusters in Fig 1 can be described as follows:

1. The ‘Patient characteristics’ cluster is at the center of the map, and it describes the characteristics of the individual patient, for example: ‘aged over sixteen’ (number 32) and ‘psychiatric history’ (number 23). Furthermore, the cluster contains general characteristics matching the MPU admission, such as diagnostic dilemma: ‘either organic explanation of disturbed behavior or psychiatric explanation of somatic symptoms’ (number 26) and ‘improvement in clinical status is expected within reasonable time frame on the MPU’ (number 30).
2. The cluster at the bottom was called ‘Patient context’. This cluster contains factors describing the interaction between patient and environment. It contains factors about the acceptability of MPU admission for both patients and family/caregivers (number 25 and 34) and also the factor ‘problematic patient-staff interaction’ (number 14). The experts decided that this cluster is different from the nearby cluster, ‘Patient characteristics’. They therefore choose a five rather than a four cluster solution.
3. The cluster on the far left in Fig 1 was called ‘Staff competencies and organizational pre-requisites’. This cluster contains the two factors that were rated as the most important: ‘competent nurses available on the MPU to meet patients’ care needs’ (number 1); and ‘medical facilities and skills on MPU, i.e. competent physicians available on the MPU to meet patients’ care needs’ (number 2). This cluster contains factors related to general availability of facilities and staff, e.g., ‘number of available beds’ and ‘number of patients per nurse’. Furthermore, some organizational characteristics were incorporated this cluster, e.g. ‘specialization of the ward’.
4. The cluster on the upper right-hand side was called ‘Medical needs and capabilities’. It contains many factors describing medical needs based on physical problems and some factors that may require specific facilities to provide good care for a patient. For example, ‘patient has been diagnosed as needing coronary care unit’ (number 73) or ‘HIV with active disease’ (number 78).
5. Lastly, the cluster on the lower right-hand side was named ‘Psychiatric symptoms and behavioral problems’ because some medical problems can be accompanied by behavioral problems without them having to be a psychiatric disorder. On the other hand, the cluster also contains many psychiatric symptoms and disorders. The three most important factors in this cluster appeared to be catatonia, suicidal behavior, and aggression.

### Utilization

To implement the five-cluster solution for an MPU admission framework, the core group defined three inclusion criteria and two exclusion criteria, based on this framework. To facilitate rapid clinical implementation, the core group advised the field to use a short questionnaire involving all five clusters. The questions are shown in Table 2. For question A, on disruptive behavior, the core group mentioned six aspects that particularly need to be addressed: agitation/aggression; suicidal behavior or deliberate self-harm; disinhibition; absconding or wandering behavior; calling out, moaning, or making other sounds; and compliance with clinician’s instructions.

**Table 2:** Short questionnaire to decide on admission of a patient to an MPU.

Inclusion
A. Is there disruptive behavior?
B. Is there a somatic reason for admission?
C. Are there no other social or professional options to deal with the situation?
Exclusion
D. Does the MPU have competent staff and sufficient/insufficient facilities?
E. Are there physical or psychiatric needs that cannot be addressed at the MPU?

Every MPU can further describe its own facilities and the needs that can and cannot be addressed at that specific MPU. The factors in the factor list (S3 Appendix) can be used to make a systematic local description. In order to admit a patient to an MPU the answers to questions A through C should be ‘Yes’, the answer to question D should indicate that the staff are competent and that there are sufficient facilities available, and the answer to question E should be ‘No’.

## Discussion

Medical Psychiatry Units (MPUs) care for complex comorbid medically and psychiatrically ill patients in a hospital setting. Medical and psychiatric acuity capabilities as well as organization of these units may vary substantially [12, 28]. The absence of research on admission criteria for MPUs hampers effective communication of diagnostic and treatment capabilities to patients and their caregivers, referrers, and payers. Furthermore, elucidating these factors will facilitate effectiveness and cost-effectiveness studies, by making MPUs comparable in terms of inclusion and exclusion criteria. This is the first study carried out to investigate the decision to admit to an MPU. We used a concept mapping approach, with the input of psychiatrists and other medical specialists, to establish factors influencing this decision, resulting in a five-cluster solution. The five clusters of criteria for the decision making on MPU admission appeared to be: 1. Staff competencies and organizational prerequisites; 2. Patient context; 3. Patient characteristics; 4. Medical needs and capabilities; and 5. Psychiatric symptoms and behavioral problems. These five clusters can be addressed by a generic, short questionnaire containing inclusion and exclusion criteria for admission to an MPU. The findings of this study enhance the ability to compare units, to perform effectiveness and cost-effectiveness analyses, and to generalize these results. This can be an important result for this field of medicine, where effectiveness and cost-effectiveness research has not significantly progressed in the past decade [21].

Kathol and colleagues developed Medical Psychiatry Units (MPUs) categories based on the level of psychiatric and medical acuity capabilities [1, 29–31]. This classification in two dimensions distinguishes four types of MPUs. Type III and IV MPUs are considered ‘high acuity’ MPUs or MPUs in the new vernacular [21], because they can handle both psychiatric and medical problems at moderate to high levels of acuity. These dimensions of psychiatric and medical acuity capabilities are clearly recognizable in the results of our current study: the availability of competent staff, adequate facilities, and the availability of medical and psychiatric care facilities are important factors for the admission decision (Table 2), mainly on the exclusion side. Psychiatric needs and capabilities did not arise as a separate cluster in the underlying concept map but were recognized as being important by the clinical expert group and, as such, was included in the questionnaire. A new finding of our concept mapping study is the incorporation of healthcare and social context, which was included in the question: ‘Are there no other social or professional options to deal with the situation?’. This question illustrates that the need for MPU admission is context-dependent. Available alternative facilities in a specific hospital, such as adequately staffed, pro-active psychiatric consultation-liaison services, delirium units, psychogeriatric wards or neurologic high care units, can change the local need for MPU admission. In an ideal world, psychiatry would be integrated fully into hospital practice, and MPUs would not be needed.

### Strengths and limitations

The systematic step-by-step concept mapping procedure, has previously been shown to be useful by making the decision processes of various health related contexts more explicit [23]. Our project was carried out with a high number of participating experts. The diversity in specialisms of the participating experts, in the core group as well as in the entire clinical expert group, and the similarity of results between specialisms, improves the generalizability of our results. The use of multiple ways for generating factors, namely an extensive list of articles and experts’ opinions, ensures that all relevant factors are included. Remarkably, the final number of clusters agreed upon (n = 5), was below the average number of clusters resulting from the sorting of all participants (average = 7.8). Although participants tended to make more distinctive clusters with very specific topics when sorting, fewer details were actually needed for the actual decision to admit a patient to an MPU, thus enabling quick and clear decision making.

A limitation of this study is that only Dutch experts participated in the concept mapping process, whereas experts from other countries may have rated the importance and commonness of factors differently, based on their specific health system contexts. However, this limitation is mitigated by conducting an extensive literature search that included articles, and thus factors, from the United States, Europe, Australia, and Japan. This Dutch conceptual model may therefore also be relevant for other countries.

It is possible that some ‘politically sensitive’ factors were rated lower when self-reported by medical specialists, while in reality these factors may subconsciously influence the admission decision. The impact of authority and the personality of participants on the rating of other participants was minimized by having participants do the ranking in private and using the results anonymously.

Finally, only physicians participated in this study, while in reality nurses influence the admission decision as well, and their competences play a key role in medical and psychiatric care abilities of MPUs [1]. This was appreciated by the participants who rated the competences of the available nurses as being more important than the competences of the physicians available. The importance of a cross-trained, skilled nursing staff with character traits such as flexibility and adaptability is emphasized extensively in literature [1, 29, 32, 33].

### Future research

A conceptual framework for the admission to high acuity MPUs has been established by this study. Future research should examine the generalizability of this Dutch framework to other countries. Furthermore, discrete choice experiments could be used to gain more insight into the trade-offs in the admission process. The actual use of our five questions for MPU admission decisions should be implemented, tested, and validated in daily practice so that the effectiveness and cost-effectiveness of these MPU facilities can be evaluated in prospective clinical designs. Effectiveness studies should include functional and disease-related outcomes, quality of life, the effect of MPUs on staff and caregivers, as well as the effect of MPUs in reducing stigma, care utilization, and costs.

### Conclusion

A broad range of factors influence the decision to admit a patient to an MPU. Using a concept mapping approach, a consensus-based conceptual framework could be developed indicating the medical and organizational factors that determine the MPU admission decision. It is expected that by implementing the five criteria derived from this conceptual framework clinicians will find it easier to make a correct, consistent, and transparent admission decision for complex patients with psychiatric and physical disorders to an MPU.

## Supporting information

S1 Appendix Summary of Dutch MPU field norms. (DOCX)

S2 Appendix Search strategy and used articles. (DOCX)

S3 Appendix Complete list of clusters and factors. (DOCX)

## Abbreviations

CIU: Complexity Intervention Unit
MDS: multidimensional scaling
MPU: Medical Psychiatry Unit
SD: standard deviation
