# Supplementary material for: Factors influencing the admission decision for Medical Psychiatry Units: A concept mapping approach
Source: PLoS One. 2019 Sep 17;14(9):e0221807. doi: 10.1371/journal.pone.0221807 (PMC6748432; doi:10.1371/journal.pone.0221807)
Supplement: S1 Appendix — (DOCX) [file pone.0221807.s001.docx]

| 1. | Acuity of the CIU corresponds to that of a regular psychiatric combined with a general medical ward. |
| --- | --- |
| 2. | a. A CIU provides 24 hours a day, 7 days a week integrated psychiatric and general medical diagnostics and treatment to inpatients;  b. The medical specialists are trained and experienced in treating patients with complex general medical and psychiatric health needs;  c. A CIU is capable of handling at least a non-limitative list of ten care needs and treatment methods. |
| 3. | Specialist medical care on the CIU is organizationally completely embedded in the relevant hospital. |
| 4. | The management and operation of a CIU should be based on both business and medical expertise and should comply with the usual organizational form of the hospital. |
| 5. | Hospitals with a CIU have both a psychiatric consultation service and an outpatient clinic for hospital psychiatry. |
| 6. | A CIU has chain care agreements inside and outside the hospital, with both referrers and aftercare agencies. |
| 7. | General medical and psychiatric nursing expertise of sufficient level is guaranteed. |
| 8. | Minimum availability of psychological and paramedical disciplines. |
| 9. | Facility design enables both psychiatric and general medical specialist care. |
| 10. | Electroconvulsive therapy is readily available for CIU-patients, 24 hours a day, 7 days per week. |

# S1 Appendix Summary of Dutch CIU field norms (12)
