# Supplementary material for: Factors influencing the admission decision for Medical Psychiatry Units: A concept mapping approach
Source: PLoS One. 2019 Sep 17;14(9):e0221807. doi: 10.1371/journal.pone.0221807 (PMC6748432; doi:10.1371/journal.pone.0221807)
Supplement: S2 Appendix — (DOCX) [file pone.0221807.s002.docx]

# S2 Appendix Search strategy and articles used for generation of statements

A search in Embase, Medline, Web of science, PsycINFO, Scopus, CINAHL, Cochrane and Google Scholar using this syntax yielded 4591 articles after deduplication.

Search strategy

(((('medical psychiatric' OR 'medical and psychiatric' OR 'medical and mental' OR 'physical and psychiatric' OR 'medical psychiatry' OR 'medicine psychiatry ' OR 'medical psychiatric' OR 'psychiatric medical' OR 'psychiatric and medical' OR 'psychiatry medical' OR 'medical mental' OR 'mixed mental' OR 'mixed psychiatric' OR 'mixed psychiatry' OR 'med psych' OR 'psych med') NEAR/6 (unit OR units OR department* OR ward OR wards OR inpatient* OR setting* OR service*))):ad,ab,ti  OR ((((joint OR joined OR mixed OR combined OR integrated) NEAR/6 (unit OR units OR ward OR wards)) OR 'medically complex' OR 'difficult medical'  OR 'complexity intervention'):ad,ab,ti AND (psychiatry/exp OR 'psychiatric department'/de OR psychiatrist/de OR (psychiatr*):ad,ab,ti)))

- Alberque, C., Gex-Fabry, M., Whitaker-Clinch, B., & Eytan, A. (2009). The Five-Year Evolution of a Mixed Psychiatric and Somatic Care Unit: A European Experience. Psychosomatics, 50(4), 354-361.
- Bruns, W., & Stoudemire, A. (1990). Development of a medical-psychiatric program within the private sector. Potential problems and strategies for their resolution. Gen Hosp Psychiatry, 12(3), 137-147.
- Buckley, P., Freyne, A., & Walshe, N. (1994). The medical-psychiatry unit: A pilot study of conjoint care within an Irish general hospital. Psychosomatics, 35(6), 515-519.
- Chang, C., Lee, Y., Yang, M., & Wen, J. (2001). Predictors of readmission to a medical-psychiatric unit among patients with minor mental disorders. Chang Gung medical journal, 24(1), 34.
- Cowart, T., & Stoudemire, A. (1989). Nursing staff development and facility design for medical-psychiatry units. Gen Hosp Psychiatry, 11(1), 36-47.
- Fava, G. A. (1987). Medical-psychiatric service. Psychotherapy and Psychosomatics, 48(1-4), 96-100.
- Fava, G. A., Wise, T. N., Molnar, G., & Zielezny, M. (1985). The medical-psychiatric unit: a novel psychosomatic approach. Psychotherapy and Psychosomatics, 43(4), 194-201.
- Fogel, B. S. (1985). A psychiatric unit becomes a psychiatric-medical unit: Administrative and clinical implications. Gen Hosp Psychiatry, 7(1), 26-35. doi:http://dx.doi.org/10.1016/0163-8343(85)90007-6
- Fogel, B. S. (1989). Med-psych units. Financial viability and quality assurance. Gen Hosp Psychiatry, 11(1), 17-22.
- Fogel, B. S., & Stoudemire, A. (1986). Organization and development of combined medical-psychiatric units: II. Psychosomatics: Journal of Consultation Liaison Psychiatry, 27(6), 417-428.
- Fogel, B. S., Stoudemire, A., & Houpt, J. L. (1985). Contrasting models for combined medical and psychiatric inpatient treatment. Am J Psychiatry, 142(9), 1085-1089.
- George, J., Adamson, J., & Woodford, H. (2011). Joint geriatric and psychiatric wards: A review of the literature. Age Ageing, 40(5), 543-548.
- Gertler, R., Kopec-Schrader, E. M., & Blackwell, C. J. (1995). Evolution and evaluation of a medical psychiatric unit. Gen Hosp Psychiatry, 17(1), 26-31.
- Goodman, B. (1985). Combined psychiatric-medical inpatient units: The Mount Sinai model. Psychosomatics, 26(3), 179-189.
- Hall, R. C., & Kathol, R. G. (1992). Developing a Level III/IV Medical/Psychiatry Unit Establishing a Basis, Design of the Unit, and Physician Responsibility. Psychosomatics, 33(4), 368-375.
- Harsch, H. H., Koran, L. M., & Young, L. D. (1991). A profile of academic medical-psychiatric units. Gen Hosp Psychiatry, 13(5), 291-295.
- Harsch, H. H., LeCann, A. F., & Ciaccio, S. (1989). Treatment in combined medical psychiatry units: an integrative model. Psychosomatics, 30(3), 312-317. doi:10.1016/s0033-3182(89)72278-7
- Hoffman, R. S. (1984). Operation of a medical-psychiatric unit in a general hospital setting. Gen Hosp Psychiatry, 6(2), 93-99.
- Kathol, R. (1986). New UI Medical Psychiatry Unit serves special patients. Iowa Med, 76(5), 217-218.
- Kathol, R. (2000). "Integrating medical and psychiatric treatment in an inpatient medical setting": Commentary reply. Psychosomatics: Journal of Consultation Liaison Psychiatry, 41(4), 367-369. doi:http://dx.doi.org/10.1176/appi.psy.41.4.367
- Kathol, R. G. (1994). Medical psychiatry units: the wave of the future. Gen Hosp Psychiatry, 16(1), 1-3.
- Kathol, R. G., Harsch, H. H., Hall, R. C., Shakespeare, A., & Cowart, T. (1992a). Categorization of types of medical/psychiatry units based on level of acuity. Psychosomatics, 33(4), 376-386.
- Kathol, R. G., Harsch, H. H., Hall, R. C., Shakespeare, A., & Cowart, T. (1992b). Quality assurance in a setting designed to care for patients with combined medical and psychiatric disease. Psychosomatics, 33(4), 387-396.
- Kathol, R. G., Krummel, S., & Shakespeare, A. (1989). Psychiatry and internal medicine resident education in an acute care medical-psychiatry unit. Gen Hosp Psychiatry, 11(1), 23-30.
- Kishi, Y., Kathol, R., Cooney, J., McKay, S., Croonquist, T., & Barrywalker, J. (1995). Comparison of patients with combined medical and psychiatric-illness treated on a type-iv medical psychiatry unit or a medicine unit. Psychosomatics, 36(2), 212-213.
- Kishi, Y., & Kathol, R. G. (1999). Integrating medical and psychiatric treatment in an inpatient medical setting: The type IV program. Psychosomatics, 40(4), 345-355.
- Koran, L. M. (1985). Medical-psychiatric units and the future of psychiatric practice. Psychosomatics, 26(3), 171+175.
- Koran, L. M., & Barnes, L. E. A. (1982). The stanford comprehensive medicine unit: Integrating psychiatric and medical care. New Directions for Mental Health Services, 1982(15), 61-73.
- Lang, P. O., Vogt-Ferrier, N., Hasso, Y., Le Saint, L., Drame, M., Zekry, D., . . . Michel, J. P. (2012). Interdisciplinary Geriatric and Psychiatric Care Reduces Potentially Inappropriate Prescribing in the Hospital: Interventional Study in 150 Acutely Ill Elderly Patients with Mental and Somatic Comorbid Conditions. Journal of the American Medical Directors Association, 13(4).
- Leue, C., Driessen, G., Strik, J. J., Drukker, M., Stockbrugger, R. W., Kuijpers, P. M., . . . van Os, J. (2010). Managing complex patients on a medical psychiatric unit: an observational study of university hospital costs associated with medical service use, length of stay, and psychiatric intervention. J Psychosom Res, 68(3), 295-302. doi:10.1016/j.jpsychores.2009.04.010
- Lu, J. H., Chan, D. K., Ong, B., Shen, Q., Reuten, S., & Ko, A. (2009). Management and outcomes of delirium in a secured, co-located geriatric and psychogeriatric unit. J Am Geriatr Soc, 57(9), 1725-1727.
- Maier, A. B., Wächtler, C., & Hofmann, P.-D. D. W. (2007). Combined medical-psychiatric inpatient units. Zeitschrift für Gerontologie und Geriatrie, 40(4), 268-274.
- Molnar, G., & Fava, G. (1983). Characteristics of medical-psychiatric unit and liaison-consultation service patients. PSYCHOSOMATIC MEDICINE, 45(1), 77-77.
- Molnar, G., Fava, G. A., & Zielezny, M. A. (1985). Medical-psychiatric unit patients compared with patients in two other services. Psychosomatics, 26(3), 193-195, 199-200, 208-199. doi:10.1016/s0033-3182(85)72874-5
- Moss, G. R., & James, C. R. (1986). Pilot study of a behavioral medicine program in a community hospital setting. Journal of Behavior Therapy and Experimental Psychiatry, 17(1), 3-9.
- Muqtadir, S., Hamann, M. W., & Molnar, G. (1986). Management of psychotic pregnantpatients in a medical-psychiatricunit. Psychosomatics, 27(1), 31-33.
- Nomura, S., Shigemura, J., Nakamura, M., Hosaka, T., Berger, D., & Takahashi, Y. (1996). Evaluation of the first Medical Psychiatry Unit in Japan. Psychiatry Clin Neurosci, 50(6), 305-308.
- Passov, V., & Rundell, J. R. (2008). Analysis of transfers from a medical-psychiatry inpatient unit to a medical-surgical unit within 48 hours of admission. Psychosomatics, 49(6), 535-537. doi:10.1176/appi.psy.49.6.535
- Porello, P. T., Madsen, L., Futterman, A., & Moak, G. S. (1995). Description of a geriatric medical/psychiatry unit in a small community general hospital. The journal of mental health administration, 22(1), 38-48.
- Regan, J., Prince, T., Wilhoite, K., Acton, R. J., Hamer, G., & Wright, A. (2005). Integrating medicine and psychiatry: Psychiatric Medical Units (PMUs). Tennessee medicine: journal of the Tennessee Medical Association, 98(9), 448.
- Stoudemire, A. (2000). Integrating medical and psychiatric treatment in an inpatient medical setting. Psychosomatics, 41(4), 366-367.
- Stoudemire, A., Brown, J. T., McLeod, M., Stewart, B., & Houpt, J. L. (1983). The combined medical specialties unit: an innovative approach to patient care. N C Med J, 44(6), 365-367.
- Stoudemire, A., & Fogel, B. S. (1986). Organization and development of combined medical-psychiatric units: Part 1. Psychosomatics, 27(5), 341-345.
- Stoudemire, A., Hales, R. E., & Thomas, C. R. (1987). Medical-psychiatry units: an economic alternative for consultation-liaison psychiatry? Psychiatric Services, 38(8), 815-818.
- Stoudemire, A., Hill, C. D., Dalton, S. T., & Marquardt, M. G. (1994). Rehospitalization rates in older depressed adults after antidepressant and electroconvulsive therapy treatment. J Am Geriatr Soc, 42(12), 1282-1285.
- Stoudemire, A., Linfors, E., Kahn, M., & Houpt, J. L. (1985). Masked depression in a combined medical-psychiatric unit. Psychosomatics, 26(3), 221-228.
- Stoudemire, G. A., & Fogel, B. S. (1988). The emergence of medical psychiatry: A provocative viewpoint. Psychosomatics, 29(2), 207-213.
- Strain, J. J. (1982). Needs for Psychiatry in the General Hospital. Hospital and Community Psychiatry, 33(12), 996-1001.
- Sullivan, M. D., Ward, N. G., & Laxton, A. (1992). The woman who wanted electroconvulsive therapy and do-not-resuscitate status: Questions of competence on a medical-psychiatry unit. Gen Hosp Psychiatry, 14(3), 204-209.
- Summergrad, P. (1994). Medical psychiatry units and the roles of the inpatient psychiatric service in the general hospital. Gen Hosp Psychiatry, 16(1), 20-31.
- Swenson, J. R., & Mai, F. M. (1992). A Canadian medical-psychiatric inpatient service. Can J Psychiatry, 37(5), 326-334.
- Tulloch, J. A. (1986). Ward for patients with disturbed behavior in a geriatric hospital - an evaluation. Australian Journal on Ageing, 5(3), 27-31.
- von Rad, M., & Sellschopp, A. (1987). The Integrated Psychosomatic Inpatient Unit. Psychotherapy and Psychosomatics, 48(1-4), 101-109.
- Wise, M. G., Rundell, J.R. (2002). Textbook of conultation-liaison psychiatry. Washington: American Psychiatric Publishing.
- Withersty, D. J., Shemo, J. P., Waldman, R. H., & Stevenson, J. M. (1980). Evaluating a conjoint psychiatric–medical inpatient unit: A one year follow-up study of depressed patients. Journal of Clinical Psychiatry.
- Wong Tin Niam, D. M., Geddes, J. A., & Inderjeeth, C. A. (2009). Delirium unit: our experience. Australas J Ageing, 28(4), 206-210.
- Young, L. D., & Harsch, H. H. (1986). Inpatient unit for combined physical and psychiatric disorders. Psychosomatics, 27(1), 53-60.
- Young, L. D., & Harsch, H. H. (1989). Length of stay on a psychiatry-medicine unit. Gen Hosp Psychiatry, 11(1), 31-35.
