# Supplementary material for: Factors influencing the admission decision for Medical Psychiatry Units: A concept mapping approach
Source: PLoS One. 2019 Sep 17;14(9):e0221807. doi: 10.1371/journal.pone.0221807 (PMC6748432; doi:10.1371/journal.pone.0221807)
Supplement: S3 Appendix — (DOCX) [file pone.0221807.s003.docx]

# S3 Appendix Complete list of clusters and factors

Three most important factors within a cluster are underlined.

**1. Staff competencies and organizational pre-requisites**

1. competent nurses available on the CIU to meet patient's care needs

2. medical coverage on CIU, i.e. competent physicians available on the CIU to meet patients care needs

3. specialization of the CIU, i.e. focus on particular pathologies

4. hospital profile, i.e. specific area of interest

5. number of CIU beds available at the time of referral/patient presentation to the CIU

6. possibility to transfer patient in case of altered psychiatric/medical care acuity

7. possibilities for aftercare

8. source of referral: internal or external

9. source of referral: from specific internal department (for example ICU)

10. pressure from the informal network

11. time of referral

12. formal agreements on admission with referrer or health care insurance

13. patient's admission is refused by other wards

15. infection isolation rooms available on the CIU

16. compulsory admission facilities available on the CIU

18. nursing staff ratio, i.e. number of patients per nurse

19. quality of psychosocial and psychiatric care at referring ward

20. alternative psychosomatic care available, for instance Consultation-Liaison Psychiatry or Geriatrics

21. distance between residential area and the hospital, limits treatment and diagnostics in outpatient setting

**2. Patient context**

14. problematic patient-staff interactions (on the referring ward)

17. required nursing care intensity of patients currently admitted to the CIU

25. acceptability of CIU admission to a patient

28. quality and availability of medical and psychiatric care in residential area

33. patient's primary support group, like family, is available

34. acceptability of CIU admission to patient's family and caregivers

**3. Patient characteristics**

22. noncompliance with (previous) treatment

23. psychiatric history

24. high number of previous hospitalizations and emergency hospital visits with unclear diagnosis

26. diagnostic dilemma: either organic explanation of disturbed behavior or psychiatric explanation of somatic symptoms

27. behavioral or psychiatric problem hampers other medical treatment

29. adverse psychiatric reaction to medical treatment that requires inpatient care

30. improvement in clinical status is expected in reasonable time frame on the CIU

31. level of self-care / (instrumental) Activities of Daily Living

32. aged <16 years

**4. Medical needs and capabilities**

73. medical telemetry of vital functions is needed

74. patient has indication for coronary care unit

75. thrombolysis is needed (i.e. because of ischemic CVA)

76. respiratory insufficiency

77. post-transplant care needed (within 1 year after transplantation)

78. HIV with active disease

79. patient needs high care (for instance cardiological, neurological or pulmonal support)

80. airborne infectious diseases

81. fulfills criteria for ICU admission

82. level of consciousness

83. burns with indication for admission on burns unit

84. patient has indication for or has received bone marrow transplantation

85. patient has indication for high dose chemo (strict isolation necessary) or phase 1 chemo

86. patient needs radiation therapy

87. patient at high risk of severe postoperative complications

88. patient needs peritoneal dialysis or hemodialysis needed

89. patient has somatic admission indication

90. patient needs somatic specialist care

**5. Psychiatric symptoms and behavioral problems**

35. agitation

36. suicidal behavior

37. aggression

38. confusion

39. deliberate self-harm

40. severe cognitive impairment

41. pica (persistent eating of nonnutritive substances)

42. depression

43. mental retardation

44. psychosis

45. obsessions and coCIUlsions

46. anxiety

47. hypochondriasis

48. mania

49. severely disturbed eating habits

50. apathy

51. personality change

52. catatonia

53. organic and alcohol hallucinosis

54. psychiatric condition linked to pregnancy, peripartum, postpartum, puerperium

55. autism spectrum disorder

56. schizophrenia and schizophreniform disorder

57. schizoaffective disorder

58. bipolar disorder

59. disruptive mood dysregulation disorder

60. major depressive disorder

61. persistent depressive disorder

62. anxiety disorder

63. obsessive-coCIUlsive disorder

64. posttraumatic stress disorder

65. adjustment disorder

66. dissociative disorder

67. somatic symptom disorder and related disorders (including conversion disorder, factitious disorder

68. feeding and eating disorder

69. substance-related and addictive disorder

70. treatment-resistant delirium

71. major neurocognitive disorder (dementia)

72. (severe) personality disorder
